# Weight cycling and relative energy deficiency in sport syndrome in an elite female muaythai athlete: a case study

**DOI:** 10.3389/fspor.2025.1599131

**Published:** 2025-05-15

**Authors:** Viktorie Bulínová, Adam Wagner, Michal Kumstát

**Affiliations:** Department of Sport Performance and Exercise Testing, Faculty of Sport Studies, Masaryk University, Brno, Czechia

**Keywords:** weight loss, athlete, female, body composition, health outcomes, combat sports

## Abstract

**Introduction:**

Weight cycling—defined as repeated, chronic, and often extreme weight loss is a widespread practice in combat sports. However, it may lead to symptoms related to Relative Energy Deficiency in Sport (REDs). This case study investigates the impact of a five-week fight camp on the health and performance of an elite female Muaythai athlete, with a particular focus on metabolic and physiological adaptations.

**Methods:**

A 23-year-old professional Muaythai fighter was monitored over a seven-week period, encompassing a control phase, a five-week fight camp, the fight week, and post-fight recovery. Measurements included body composition, resting metabolic rate (RMR), blood biomarkers, and performance in a cycling power test. Dietary intake and training load were also tracked.

**Results:**

The athlete's RMR decreased by 253 kilocalories per day. Blood markers indicated increased creatinine and urea levels, along with unfavorable changes in lipid profile. Maximum cycling power output decreased by 27%. Despite a significant energy deficit, fat-free mass loss was limited to 0.6. These findings suggest metabolic adaptations, signs of renal strain, and symptoms of REDs.

**Conclusion:**

Weight cycling induced physiological and metabolic adaptations consistent with REDs, negatively affecting health and performance. The results highlight the importance for individualized and evidence-based weight management strategies to mitigate negative health outcomes and enhance athletic performance in combat sports.

## 1 Introduction

In many combat sports, including boxing, judo, and Muaythai, athletes are categorized into weight classes, aligning competitors based on their weight to minimize the risk of injuries resulting from a significant weight advantage (1). The primary motivation behind manipulating body weight in these sports is to meet the weight limit of a specific weight class. It is common for athletes to compete in a weight class below their usual training body weight, employing both chronic weight loss (CWL) and rapid weight loss (RWL) strategies (1).

CWL involves sustaining an energy deficit over time, with the duration instated by the required weight loss (2). During the fight week (FW), athletes implement RWL strategies, that can result in body weight reduction up to 10%. These methods include fluid intake restriction, sodium reduction, glycogen store depletion, low-residue diets, and active and passive sweating. The extent of CWL and RWL implementation often depends on the interval between official weigh-ins and competition, which varies across amateur and professional settings (3–6).

Health risks associated with RWL include increased cortisol levels, hyponatremia, impaired immune function, and elevated creatinine levels, indicating acute kidney injury (7). Dehydration during RWL is linked to reduced plasma volume, increased blood viscosity, and heightened cardiovascular strain, raising the risk of cardiovascular problems. Moreover, RWL can negatively affect the mental health, leading to short-term declines in memory, concentration, self-esteem and an increase in confusion, depression, and aggression (4, 8, 9). The frequency of RWL strategy use is also associated with a higher incidence of injuries among fighters (10).

Despite known health risks and potential performance impairments, many athletes perceive the competitive advantage gained through weight cutting to outweigh the drawbacks (11). Evidence shows that RWL can impair performance, particularly when recovery time post-weigh-in is less than one hour (12). However, research by Mendes (13) and Cengiz (14) suggests that adequate recovery time can help mitigate these adverse effects.

On the other hand, CWL is linked with the risk of developing Relative Energy Deficiency in Sport syndrome (REDs) (15). REDs reflect adverse physiological effects observed in athletes when their energy intake does not adequately cover the body's functional needs after subtracting training or competition energy expenditure. Its effects include menstrual, endocrine, metabolic (e.g., reduced resting metabolic rate), hematological (e.g., elevated creatinine and urea), psychological, cardiovascular, gastrointestinal, and immunological disturbances (16). These consequences impair training adaptations and performance by reducing endurance capacity, increasing injury risk, lowering training adherence, impairing coordination, depleting glycogen stores, suppressing muscle protein synthesis, and reducing muscle strength (16). Case studies by Kasper (7) and Langan-Evans (17) provide vivid examples of REDs during fight preparation.

Over the past two decades, considerable research has explored the physiological and psychological consequences of weight manipulation in combat sports. Studies across nutrition, exercise physiology, and sports psychology have documented the prevalence, methods, and effects of weight-cutting strategies, establishing a solid knowledge base (18, 19). Seminal contributions—such as those by Franchini (11) and Artioli (12), have detailed the widespread use of RWL and its negative consequences on athlete health and performance. However, prior research has largely overlooked how these practices affect specific athlete profiles, especially in underrepresented populations such as female fighters (19). The current study addresses this gap by contextualizing weight-cutting strategies within the complex training framework of an elite female athlete.

In recent years, there has been growing recognition of the health and performance risks linked to body weight manipulation in combat sports (7, 17). This case study adds to current knowledge by examining a female Muaythai athlete and how weight-cutting strategies affect her health and performance.We hypothesize that combining chronic weight loss (CWL) and rapid weight loss (RWL) has a negative impact on female athletes. RWL may increase the risk of acute complications such as dehydration, cardiovascular strain, and cognitive issues (7, 17), while CWL may lead to longer-term problems, including REDs (16, 17). These effects likely depend on the time available for recovery between weigh-in and competition.

This study aims to explore how CWL and RWL strategies contribute to the development of REDs through monitoring physiological, metabolic, and performance changes during a structured fight camp in a female Muaythai athlete.

## 2 Materials and methods

### 2.1 Athlete overview

The subject was a 23-year-old professional female Muaythai athlete (57.5 kg, 1.65 m) diagnosed with hypothalamic secondary amenorrhea. Since starting her competitive career at 18, she has participated in 48 bouts (22–13 amateur, 8–5 professional). Based on the McKay et al. classification (20), she qualifies as a Tier 4 athlete. She typically competes 8–10 times per year in the flyweight (>51 kg) and bantamweight (>54 kg) categories, following consistent weight-loss patterns. Prior to the study, she had been training six times weekly for three months (4 Muaythai sessions, 2 strength training sessions).

### 2.2 Case report design and overview of nutritional and training intervention

The athlete underwent a simulated fight preparation to reduce body mass for the bantamweight category (<54 kg), requiring a > 3.5 kg (>6%) weight loss over five weeks. The preparation for the simulated fight mirrored that of a real fight, with the female athlete participating in a fight camp aimed at reducing her body weight. The follow-up period included one control week and one post-match week for a total follow-up period of 7 weeks.

Instead of an actual bout, a critical performance test was used as a simulated fight (SF), occurring four hours post weigh-in (WI) under amateur conditions. The objective was to closely approximate the natural conditions of a real fight, with the female athlete undergoing a critical power test at a time corresponding to when a real fight would occur. The total observation period was 7 weeks, divided into four phases of varying durations of several weeks (WK) or days (D).

**Phase 1:** Control week (CW) 7D—general training regime when the fighter is not in preparation for a fight.

**Phase 2:** Fight camp diet (FCD) 28D (−5WK, −4WK, −3WK, −2WK)—training regime focused on weight reduction and specific skills for a fight.

**Phase 3:** Fight week (FW) 4D (−1WK, WI + SF)- final weight loss using RWL strategies and reduced training load and weigh-in + simulated fight.

**Phase 4:** Post-fight week (PFW) – 7D (+1WK) – a more relaxed eating and training regime).

The methodology further elaborates on the measurements used.

**Phase 1:** During Phase 1 CW, the female athlete followed a diet *ad libitum*. The average daily energy intake was 2 198 ± 128kcal (Table 1). Macronutrient breakdown included 72 ± 10 g of fat (1.3 g/kg/d), 285 ± 33 g of carbohydrates (5 g/kg/d), and 104 ± 6g of protein (1.8 g/kg/d) during this phase. Fiber intake in Phase 1 was 26 ± 5 g/d. The female athlete's weekly training schedule (refer to Table 2) included one aerobic continuous bike ride (BR), two resistance training sessions (RT), three Muaythai training sessions (MT), and one other training session (OT). The aerobic BR involved a 60-min bicycle ride at a cycling speed corresponding to FATpeak. RT comprised a whole-body workout with general strength/speed exercises concurrently performed in a superset, incorporating speed/strength, ballistic, and reactive strength modalities. Volume loads and intensities were determined through 1 repetition maximum (RM) testing. MT training was specific sports training lasting 60–90 min, and OT involved sports activity at college (the athlete is a student).

**Table 1:** Energy intake, expenditure, and availability across all study phases (mean ± SD).

Phase	EI (kcal/day)	DIT (kcal/day)	NEAT (kcal/day)	EEE (kcal/day)	24-h EB (kcal/day)	EA (kcal/kg FFM/day)
Control week	2,198 ± 138	220 ± 14	n/a	304 ± 317	n/a	42 ± 6
−5 WK	1,541 ± 30	154 ± 3	238.0 ± 96.0	393 ± 279	−792.0 ± 404.0	25 ± 6
−4 WK	1,557 ± 39	156 ± 4	303.0 ± 67.0	381 ± 240	−712.0 ± 262.0	26 ± 5
−3 WK	1,496 ± 43	149 ± 4	249.0 ± 104.0	387 ± 244	−945.0 ± 246.0	25 ± 6
−2 WK	1,506 ± 34	151 ± 3	241.0 ± 23.0	353 ± 241	−845.0 ± 253.0	26 ± 6
−1 WK	1,371 ± 65	137 ± 7	298.0 ± 50.0	380 ± 36	−1,034.0 ± 161.0	22 ± 1
+1 WK	2,096 ± 107	210 ± 11	n/a	354 ± 51	n/a	41 ± 3

EI, energy intake; DIT, diet-induced thermogenesis; NEAT, non-exercise activity thermogenesis; EEE, exercise energy expenditure; 24-h EB, 24-hour energy balance; EA, energy availability (per kg of fat-free mass); WK, week.

Values for Control Week and +1 WK may not include NEAT and EB due to technical limitations in data collection.

**Table 2:** Training schedule.

Type of training	Phase 1	Phase 2				Phase 3		Phase 4
	.−6 WK	.−5 WK	.−4 WK	.−3 WK	.−2 WK	.−1 WK	WI + F	. + 1 WK
Muaythai	3	3	4	4	4	3	1	4
Strenght	2	2	3	3	2			
Psychomotoric		1	1	2	2			
Intervals		1	1	1				
Aerobic	1	1		1	1			
Other	1	1						1
Total	7	9	9	11	9	4		5

WK, week; WI, weigh-in, F, fight. Phase 1 – control week, Phase 2 - fight camp diet, Phase 3 - fight week and Phase 4 - post-fight week.

**Phase 2:** The female athlete followed a fight camp diet based on the “3,2,1” principle, entailing macronutrient intake of 3 g/kg/d for carbohydrates, 2 g/kg/d for proteins, and 1 g/kg/d for fats (2, 21). This dietary approach is a standard practice for the athlete in preparation for a fight. The energy intake during this phase averaged 1,525 ± 29 kcal per day (Table 1). Macronutrient distribution included 50 ± 2 g of fat (0.8 g/kg/d), 170 ± 4 g of carbohydrates (3 g/kg/d), and 104 ± 3 g of protein (1.8 g/kg/d). In Phase 2, the fiber intake was 21 ± 2 g. A detailed description of −5 WK to −2 WK is provided in Table 1. The athlete's weekly training schedule (Table 2) comprised one aerobic continuous bike ride (BR), two resistance training sessions (RT), three Muaythai training sessions (MT), one Interval training (IT), one psychomotor training (PT), and one other training session (OT). PT involved exercises with a tennis ball (juggling, catching the ball in punches, etc.) for 30 min. IT on the assault bike included a 5–10-minute warm-up, followed by 6 × 5 min at 90% HR max (rest between intervals 2 min), and a 5-minute cool down (total training time 50–60 min).

**Phase 3:** In the fight week, the female athlete incorporated rapid weight loss (RWL) strategies such as glycogen store depletion, low fiber intake, and low sodium intake over a 4-day duration (−1 WK). The energy intake averaged 1 371 ± 65kcal per day (Table 1). Macronutrient breakdown included 92 ± 5 g of fat (1.6 g/kg/d), 62 ± 10 g of carbohydrates (1.1 g/kg/d), and 85 ± 10 g of protein (1.5 g/kg/d). In Phase 3, the fiber intake was 7 ± g. A detailed description of −1 WK is outlined in Table 3. The athlete's weekly training schedule (Table 2) included three Muayhai training sessions.

**Table 3:** Within-day energy balance calculation.

Date		Intake	Expenditure				Hourly calculations	EB hr by hr
	Time	EI	DIT	NEAT	EEE	BMR	TEE hr to hr	
24.05.2021	0:00–1:00	0	0	0	0	64	64	−64
	1:00–2:00	0	0	0	0	64	64	−64
	2:00–3:00	0	0	0	0	64	64	−64
	3:00–4:00	0	0	0	0	64	64	−64
	4:00–5:00	0	0	0	0	64	64	−64
	5:00–6:00	0	0	0	0	64	64	−64
	6:00–7:00	0	0	0	0	64	64	−64
	7:00–8:00	0	0	11	0	64	74	−64
	8:00–9:00	0	0	33	0	64	97	−64
	9:00–10:00	261	26	11	0	64	100	161
	10:00–11:00	0	0	35	0	64	99	−99
	11:00–12:00	405	41	55	0	64	160	245
	12:00–13:00	0	0	31	0	64	94	−94
	13:00–14:00	0	0	2	300	64	366	−366
	14:00–15:00	0	0	45	0	64	108	−108
	15:00–16:00	300	30	23	0	64	117	−117
	16:00–17:00	0	0	9	0	64	73	−73
	17:00–18:00	0	0	24	0	64	88	−88
	18:00–19:00	0	0	0	590	64	654	−654
	19:00–20:00	561	56	27	0	64	146	415
	20:00–21:00	21	2	1	0	64	67	−65
	21:00–22:00	0	0	0	0	64	64	−64
	22:00–23:00	0	0	0	0	64	64	−64
	23:00–24:00	0	0	0	0	64	64	−64
	24-h Total	1,548	155	307	890	1,530	2,882	−1,609

EI, energy intake; DIT, diet-induced thermogenesis; NEAT, non-exercise activity; EEE, energy exercise expenditure; BMR, basal metabolic rate; TEE, total energy expenditure; and EB, energy balance, Phase 1 – control week, Phase 2 - fight camp diet, Phase 3 - fight week and Phase 4 - post-fight week.

After the weigh-in (4-hour period), the energy intake was 1,013 kcal. Macronutrients consisted of 12 g of fat (0.2 g/kg), 205 g of carbohydrates (3.8 g/kg), and 15 g of protein (0.3 g/kg), with a fiber intake of 2 g. Fluid intake was 2.2 liters, and sodium intake was 2.24 g.

**Phase 4:** In the post-fight week, the female athlete consumed food and fluids *ad libitum*. The energy intake averaged 2 093 ± 106 kcal per day (Table 1). Macronutrient distribution included 70 ± 11 g of fat (1.2 g/kg/d), 263 ± 22 g of carbohydrates (4.7 g/kg/d), and 107 ± 12 g of protein (1.8 g/kg/d). In Phase 4, the fiber intake was 20 ± 4 g. The athlete's weekly training schedule (Table 2) included four Muaythai training sessions (MT) and one other training session (OT).

Throughout all phases, no dietary supplements (except habitual coffee consumption) were implemented or allowed to limit potential ergogenic effects on subsequent performance-based testing.

### 2.3 Athlete assessment, assessment of training load, energy availability, within-day energy balance and weekly accumulated energy deficit

The athlete underwent a comprehensive assessment protocol across all phases of the study. All measurements and sampling were conducted in the fasted state, with the exception of a small carbohydrate-containing snack consumed 20 min before the critical power test. To improve clarity and allow effective tracking of physiological changes, assessed variables were categorized into four key domains:

• Morphological assessment:

Body mass (BM), body fat percentage (BF%), fat-free mass (FFM), and fat mass (FM), assessed using dual-energy x-ray absorptiometry (DXA; QDR Series Horizon, Hologic Inc.) and bioelectrical impedance analysis (InBody 770). DXA values were also used to calculate energy availability (EA).

• Functional performance assessment:

Maximal, average, and minimal power output during a 4-minute cycling test (Lode Excalibur Sport), along with derived indices such as relative power, fatigue rate, and total work, used to evaluate anaerobic performance and neuromuscular fatigue.

• Physiological and metabolic profiling:

Resting metabolic rate (RMR) was assessed via indirect calorimetry (Cortex Metalyzer 3B-R3), RMR prediction (RMR_pred_), following Compher et al. (22), and was calculated using the Cunningham equation (23). A broad panel of blood biomarkers was analysed (24, 25). These included renal markers (creatinine, urea), plasma osmolality, glucose, insulin, thyroid hormones (TSH, fT3, fT4), IGF-1, lipid profile (total cholesterol, LDL, HDL, triglycerides), iron metabolism indicators (ferritin, transferrin, soluble transferrin receptor), bone turnover markers (vitamin D, Beta-cross laps, P1NP), inflammatory marker (C-reactive protein, CRP), and sex hormones (FSH, LH, estradiol, prolactin, SHBG, testosterone). Blood samples were collected via venipuncture and analysed in a certified laboratory using standardized procedures.

Blood samples were collected via venipuncture and analysed in a certified laboratory using standardized procedures.

• Nutritional and energy availability domain:

Daily dietary intake was recorded by the athlete over 46 days, covering all phases of the study: Phase 1 (7 days), Phase 2 (28 days), Phase 3 (4 days), and Phase 4 (7 days). Records were tracked via a mobile nutrition app under supervision and analyzed using NutriPro software. Energy intake (EI), diet-induced thermogenesis (DIT), exercise energy expenditure (EEE), non-exercise activity thermogenesis (NEAT), and within-day energy balance (24-h EB) were calculated for each phase. Energy availability (EA) was determined relative to fat-free mass based on DXA-derived values. Physical activity and training load data were collected using wearable devices (ActiGraph wGT3X-BT, Garmin HRM-Run 2).

**Figure 1:**
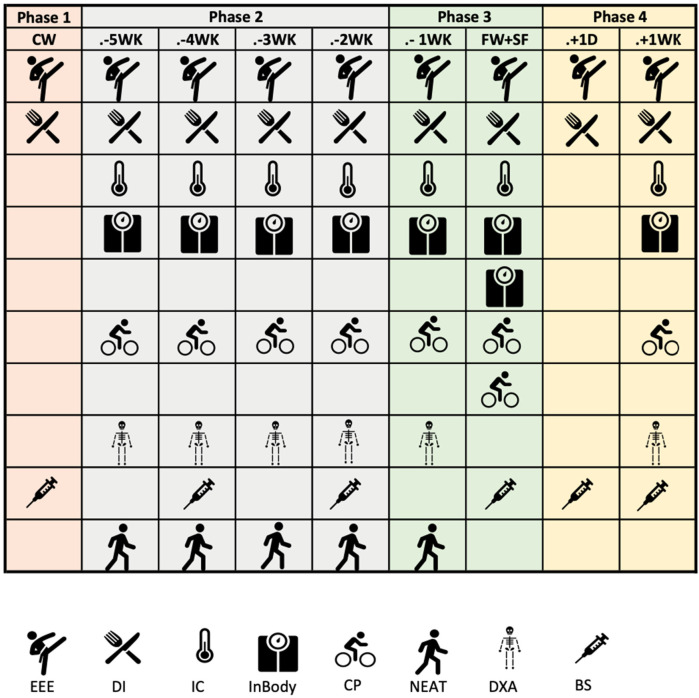
Instruments and parameters measured during the intervention. EEE, exercise energy expenditure; DI, dietary intake; IC, indirect calorimetry; CP, critical power test; NEAT, non-exercise energy expenditure; DXA - by dual-energy x-ray absorptiometry and BS, blood samples.

A summary of the measurements for each phase is illustrated in Figure 1.

### 2.4 Ethical considerations

This study was approved by the Masaryk University Research Ethics Committee (EKV-2021-004). The participant provided written informed consent prior to data collection.

## 3 Results

### 3.1 Changes in anthropometric measurements

**Figure 2:**
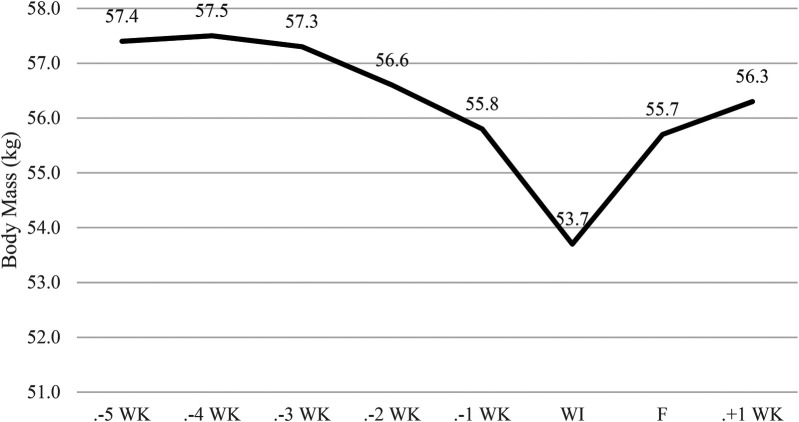
Changes in body mass. WK, week; WI, weigh-in, and F, fight. Phase 1 – control week, Phase 2 - fight camp diet, Phase 3 - fight week and Phase 4 - post-fight week.

**Figure 3:**
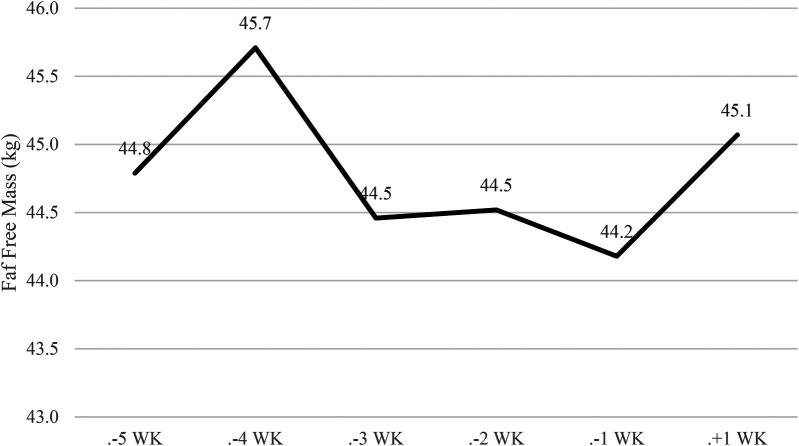
Changes in fat-free mass. WK, week; WI, weigh-in, and F, fight. Phase 1 – control week, Phase 2 - fight camp diet, Phase 3 - fight week and Phase 4 - post-fight week.

**Figure 4:**
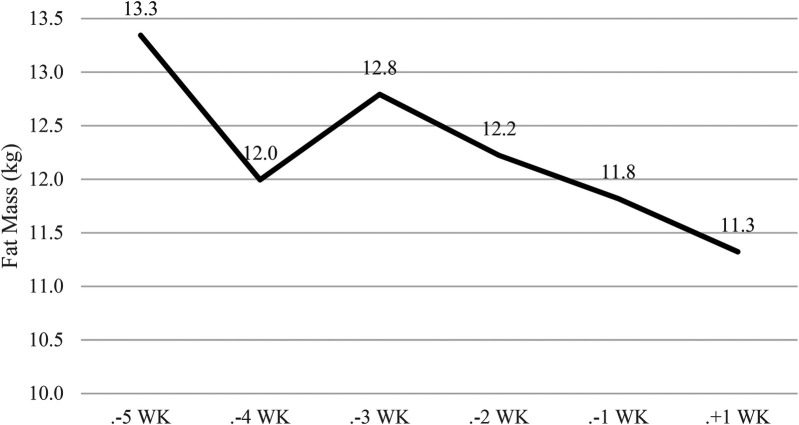
Changes in fat mass. WK, week; WI, weigh-in, and F, fight. Phase 1 – control week, Phase 2 - fight camp diet, Phase 3 - fight week and Phase 4 - post-fight week.

Figures 2–4 illustrate the temporal alterations in body mass (BM), fat mass (FM), fat-free mass (FFM), and body fat percentage (BF%). The athlete achieved the designated limit for the selected bantamweight weight category for the simulation fight, recording an official weight of 53.7 kg at 9:00 AM.

Overall, there was a −3.7 kg (<6.4%) reduction in body weight, as depicted in Figure 2. This reduction comprised −0.6 kg (<1%) in FFM (Figure 3) and 1.8 kg (<3.1%) in FM (Figure 4) during Phase 2. Despite a body mass loss of 1.6 kg during Phase 2, the athlete still needed to shed an additional 1.8 kg in the subsequent 4 days.

In Phase 3 (fight week), body weight was further reduced by −2.1 kg (<3.8%) over 4 days. A 4-hour time delay between the weigh-in and the simulation fight (01:00 PM) allowed the athlete to regain 2 kg (>3.6%).

During the post-competitive recovery period in Phase 4, the athlete experienced an increase in BM by 2.6 kg (>4.8%), FM decreased by −0.5 kg (<0.9%), and FFM increased by 0.9 kg (>1.72%).

### 3.2 Assessment of low EA on markers of female athlete triad and REDs

**Figure 5:**
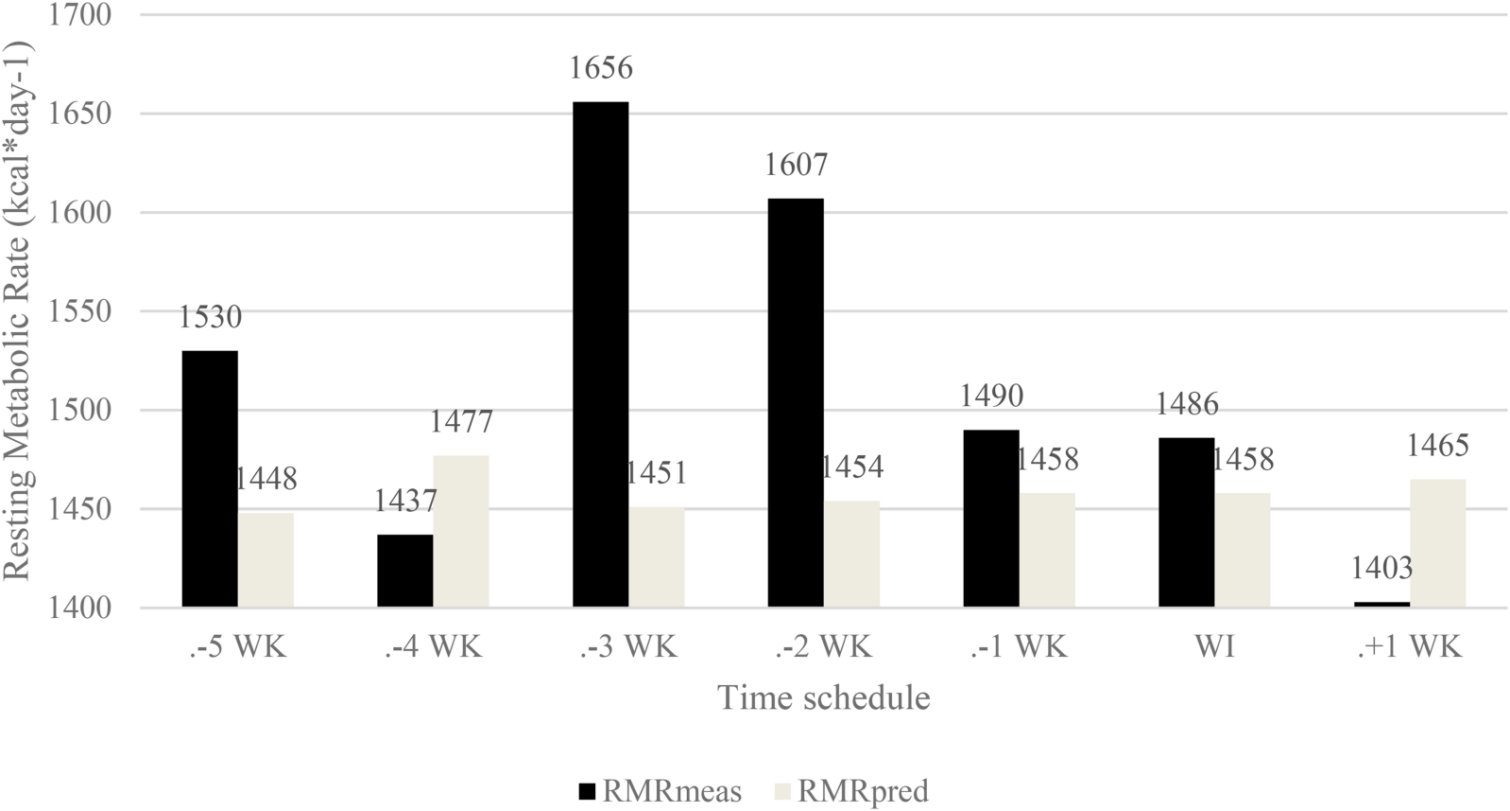
Resting metabolic rate and predicting resting metabolic rate. RMEmeas, resting metabolic rate measured; RMRpred, resting metabolic rate predicted (Cunningham); WK, week; WI, weigh in, and F, fight.

At baseline, the athlete exhibited one established symptom of the Female Athlete Triad and REDs -hypothalamic amenorrhea. Therefore, the menstrual cycle phase did not influence the data. The assessment of the athlete's RMR is presented in Figure 5. In Phase 2, there was a reduction in RMR measured (RMRm) values by −93 kcal·d−1 at −4 WK, followed by an increase of +219 kcal·d−1 at −3 WK, a reduction of −49 kcal·d−1 at −2 WK, and −117 kcal·d−1 at −1 WK. Throughout Phases 3 and 4, there was a gradual reduction in RMRm values by −4 kcal·d−1 at -WI and −83 kcal·d−1 at +1 WK, resulting in an overall reduction from baseline of −127 kcal·d−1. Concerning RMRratio [RMRm vs. RMRpredicted—calculated by Cunningham (23)], values increased compared to RMRratio, with only −4 WK (0.97) and +1 WK (0.96) measurements showing a decrease compared to RMRratio. The reduction in RMRm in Phase 4 may result from dietary intervention during Phases 2 and 3, especially in Phase 3, where the average EA was 22 kcal·kg FFM-1 ·d-1.

Table 4 displays parameters observed during the 4-minute critical power test. Unfortunately, measurements from the beginning of Phase 2 are unavailable. For the −5 WK measurements, the bicycle ergometer VO2max test was initially chosen but was later replaced by the −4 WK critical power test, more specific to the athlete's sport. The critical power test (−4 WK) initially lasted 3 min, and due to an unclear indication of typical stress test fatigue, it was extended to 4 min. The athlete experienced a decrease in maximal power output throughout the observation period, with the highest value recorded at −3 WK (392 W). It is presumed that the values measured during the −5 WK and −4 WK periods would have been even higher. There was a decrease in maximum power, with the lowest values measured in Phase 3 (285 W). The highest level of fatigue was observed in Phase 3 WI (70.12), when the athlete was dehydrated and had reduced glycogen stores after using RWL strategies.

**Table 4:** Maximal power, average power, minimal power, relative average power, relative maximal power, the tendency to fatigue, fatigue rate, and total work.

Critical power	Phase 2		Phase 3			Phase 4
	.−3 WK	.−2 WK	.−1 WK	WI	F	. + 1 WK
Duration	240,00	240,01	240,01	240,01	240,00	240,01
Maximal power	392,63	313,66	294,03	302,76	285,08	304,49
Avarage power	204,51	200,28	192,00	169,36	192,73	194,37
Minimal power	159,89	145,87	140,38	90,47	128,12	146,78
Avarage power/body weight	3,57	3,50	3,35	3,15	3,46	3,45
Maximal power/body weight	6,85	5,47	5,13	5,64	5,12	5,41
Tendency to fatigue	1,05	5,48	3,28	1,23	0,84	2,23
Fatigue rate	59,28	53,49	52,26	70,12	55,06	51,79
Total work	49,04	48,07	46,04	40,61	46,22	46,61

For the −5 WK measurements, the bicycle ergometer VO2max test was initially chosen but was later replaced by the −4 WK critical power test. The critical power test (−4 WK) initially lasted 3 min, and due to an unclear indication of typical stress test fatigue, it was extended to 4 min. WK, week, WI, weigh-in, and F, fight, Phase 2 - fight camp diet, Phase 3 - fight week and Phase 4 - post-fight week.

Table 5 details changes in biochemical parameters, with values outside reference limits highlighted in bold. Urea levels increased in the −2 WK and WI + SF periods, while creatinine levels increased in the −4 WK, −2 WK, WI + SF, and +1 D periods. Plasma osmolality was elevated (296 nmol/kg) during the V + Z period when the athlete was dehydrated due to Rapid Weight Loss (RWL) strategies. Insulin levels were within normal limits, except in the +1 D period when insulin levels were 2.5 mmol/L (indicating hypoglycemia). Total cholesterol and LDL values were elevated compared to reference values at the study's beginning and increased during the study, reaching their highest levels in the WI + SF period. Thyroid hormone levels gradually decreased, with the lowest TSH (3.681 ->2.075 mU/L) and fT3 (3.7 ->2.7 pmol/L) values in the WI + SF period, and fT4 (10.1 ->9.3 pmol/L) in the +1 D period. Vit. D values were suboptimal initially. Elevated Beta-cross laps and P1NP values at the study's beginning indicated increased bone turnover, possibly linked to the training intervention.

**Table 5:** Changes in blood clinical chemistry.

Basic biochemistry	Phase 1	Phase 2		Phase 3	Phase 4		avrg.
	CW	.−4 WK	.−2 WK	WI + F	. + 1 D	. + 1 WK	
Na (mmol/L)	140	142	141	144	141	139	136–145
K (mmol/L)	4,8	5	4,7	4,2	4,5	4,7	3,5–5,2
Cl (mmol/L)	106	106	107	**110**	107	104	98–107
Urea (mmol/L)	6	7,3	**7,5**	**11,8**	6,9	8,5	2,5–7,4
Creatinine (*μ*mol/L)	74	**82**	**104**	**99**	**90**	75	44–80
CRP (g/L)	<1	<1	<1	<1	<1	<1	0,0–5,0
Total protein (g/L)	74,4	71,7	76	80	68	69	64,0–83,0
Albumin (g/L)	48	47	49	51	43	45	35–52
P-osm (nmol/kg)	291	292	291	**296**	286	293	275–295
Diabetology							
Plasma glucose (mmol/L)	5,2	4,5	4	4,4	4,8	4,7	3,5–5,6
Insulin	x	2,9	3,5	3,4	2,5	2,7	2,6–25,0
Lipid profiles							
Total cholesterol (mmol/L)	**5,6**	**5,4**	5,7	**6,2**	**5,2**	**5,4**	2,9–5,0
HDL (mmol/L)	1,9	1,9	2,2	2	1,9	1,8	1,2–2,7
LDL (mmol/L)	**3,6**	**3,5**	3,5	**3,9**	**3,3**	**3,5**	1,2–3,0
Triglyceride (mmol/L)	0,63	0,61	0,52	0,72	**0,44**	0,68	0,45–1,70
Iron metabolism							
Sol.transferin receptor (mmol/L)	x	16,5	17,2	18,3	13,8	15,4	0,0–21,0
Transferrin (g/L)	x	2,76	2,9	2,95	2,44	2,55	2,0–3,6
Ferritinin (μg/L)	16,3	19,8	15,9	15,5	16,9	14,8	4,6–204,0
Thyroid gland							
TSH (mU/L)	3,681	2,392	2,619	2,075	2,544	3,328	0,270–4,20
T4 free (pmol/L)	10,1	10	10,4	10,1	9,3	10,1	9,0–19,0
T3 free (pmol/L)	3,7	2,9	2,8	2,7	2,8	3,5	2,6–5,7
Cardiac markers							
CK (μkat/L)	2,54	2,36	2,21	2,39	1,89	2,54	0,20–2,85
Bone turnover							
vit. D (nmol/L)	**44,4**	**45,5**	**57,5**	**60,9**	**53,4**	**56**	75–200
Beta-crosslaps (hg/L)	**907**	**838**	**708**	**777**	**703**	**631**	0–573
P1NP (μg/L)	**97**	**94**	**91**	74	**81**	**90**	15–74
Hormones							
FSH (U/L)	x	6,1	3,7	4,3	4,1	6,3	
LH (U/L)	2,7	2,3	0,3	0,4	0,3	1	
Estradiol (pmol/L)	<34	<34	<34	<34	<34	87	
Prolactin (mlU/L)	373,1	418,9	255,6	152	329,4	324,8	109–557
SHBG (nmol/L)	47,5	47,1	53,8	54,1	48,1	44,5	26–110
Cortisol (nmol/L)	525	**557**	**541**	501	461	**544**	101–536
IGF-1 (μg/L)	188	218	180	175	151	203	103–326
Testosterone (nmol/L)	x	0,93	1,09	0,97	0,86	1,05	0,29–1,67

CW, control week; WK, week; WI, weigh in; F, fight; Na, sodium; K, calcium; Cl, chloride; CRP, c-reactive pro-tein; P-osm, plasma osmolality; HDL, high-density lipoprotein; LDL, low-density lipoprotein; TSH, thyroid stimulating hormones; T4, thyroxine; T3, triiodothyronine; CK, creatine kinase; vit. D, vitamin D; P1NP, type 1 procollagen amino terminal peptides; FSH, follicle stimulating hormone; LH, luteinizing hormone; SHBG, sex hormone binding globulin, and IGF-1, insulin-like growth factor. avg., average, Phase 1 – control week, Phase 2 - fight camp diet, Phase 3 - fight week, and Phase 4 - post-fight week.

## 4 Discussion

This case study aimed to investigate the impact of the fight camp diet on health and performance parameters associated with REDs. Throughout the five weeks of low EA during the fight camp (mean daily value during Phase 2: 25.4 kcal·kg FFM-1 ·d-1 and Phase 3: 22.4 kcal·kg FFM-1 ·d-1), weight loss coincided with manifestations of REDs. Health consequences included a reduction in RMR values by −253 kcal, with the most significant drop observed one week after the low EA (LEA) period. The decrease in basal metabolism associated with REDs has also been observed in Australian female athletes (26) and fighters (7, 17, 19). Furthermore, a study on Norwegian athletes identified several markers of REDs, including reduced basal metabolism (27). In terms of lipid profile, an increase in total cholesterol (+10%) and LDL (+8%) was noted, potentially influenced by Rapid Weight Loss (RWL) strategies in the last three days.

The athlete's performance declined, with a −27% reduction in maximal and a −6% reduction in average performance during the critical performance stress test. It is hypothesized that the decline would have been more pronounced if performance results were available before the LEA. Decreased performance was also noted in the fighters in the study by Barley et al. (28). Contrary to expectations based on the REDs syndrome definition by Mountjoy (16), the study revealed relatively moderate and less significant health changes in female athletes. Further research is needed to establish the critical lower limit for energy availability.

Despite the LEA during the fight camp, a modest FFM reduction of −0.6 kg occurred. This phenomenon is attributed to the inclusion of strength training and a protein intake of 2 g/kg Body Weight (BW), supported by previous research indicating that increased protein intake and strength training can maintain muscle protein synthesis rates under low energy availability (29). The reduction in FFM during fight camp was in the Kasper (7) and Evans (17) studies.

The cumulated energy deficit during Phase 2 was −23,058 kcal, theoretically leading to an expected post-phase weight loss of −3.3 kg. However, the real loss of adipose tissue measured by DXA was only −1.5 kg, partially explained by the athlete's history of attending multiple fight camps annually and her relatively low body fat percentage.

During Phases 2 and 3, markers of creatinine and urea increased, consistent with a study involving wrestlers who experienced similar increases during RWL strategies combined with high training loads (30, 31). Elevated values of these markers suggest acute kidney injury (AKI), emphasizing the need for education on avoiding RWL and maintaining adequate hydration in athletes.

The critical performance test during the fight week exhibited positive changes, with the female athlete's performance at −1 WK being restored between the weigh-in and the fight. However, from an overall perspective, negative changes persisted, with the maximum performance of −3 WK being 25.3% higher than in the fight week. The decrease in power output during LEA, affecting glycogen stores and, subsequently, power output, is undesirable in combat sports, where peak performance is crucial during a fight. The results of the critical power test measured during fight week are similar to a study by Mendes (13), where they measured the effect of RWL use on fighters' performance. The fighters reduced BW by 5% over 5 days after a 4-hour window after weigh-ins to rehydrate their bodies and replenish nutrients. The study showed no negative effect of RWL on intermittent high-intensity exercise.

A comparison with studies by Kasper (7) and Langan-Evans (17) indicates that the unique conditions in this case study provided ecologically valid settings. Real-life training was simulated as closely as possible, allowing immediate post-weigh-in and pre-fight testing. The findings highlight the need for further research to understand the complex interactions between diet, training, and performance in combat sports.

The study showed that RWL and CWL strategies led to the development of RED syndrome symptoms during fight camp. Specifically, these included a decrease in BMR values, an increase in urea and creatine levels, a decrease in critical performance test performance, and a calculation of energy intake. Markers related to basal metabolic rate, bone density, anthropometric indices, T3 levels, sex hormones, and calculated energy intake are often used to diagnose REDs (16, 32).

This case study, despite yielding helpful information about the physiological and performance implications of weight cycling in a female Muaythai athlete, is hampered by its single-subject design. Findings cannot be generalized to all female combat sport athletes due to variability among individuals in training history, physiological adaptation, and prior experience with weight-cutting protocols. Absence of a control group constrains inference regarding separation of fight camp protocol-induced adaptations from others due to other external or cumulative factors of training. In addition, while test fight conditions were attempted to replicate competitive settings in life, absence of actual fighting may have impacted psychological and physiological responses. Lastly, the limited length of post-fight observation limits the comprehension of long-term recovery and potential late effects of decreased energy availability. Increased sample sizes, variability, and monitoring time posts-competition in future studies would be advantageous.

## 5 Conclusions

This case study explored the effects of a five-week fight camp diet characterized by low EA (LEA) on health and performance parameters in the context of REDS of a female Muaythai athlete. The athlete experienced weight loss, with notable manifestations such as reduced RMR and altered lipid profiles. Performance metrics indicated a decline in maximal and average performance during a critical performance stress test. Contrary to expectations, the study revealed relatively moderate health changes, emphasizing the need for further research to establish critical lower limits for energy availability in combat sports.

Despite low energy availability (LEA), the athlete experienced only a minimal decline in FFM, likely due to the incorporation of targeted resistance training and sufficient protein intake throughout the intervention. Notably, the calculated energy deficit exceeded the observed adipose tissue reduction, suggesting the influence of individual moderating factors, including prior experience with repeated weight cuts and inherently low body fat levels.

Elevations in serum creatinine and urea during the RWL phase indicate transient renal stress, highlighting the physiological risks associated with aggressive weight-cutting strategies and reinforcing the importance of proper hydration protocols. Although partial performance recovery was observed following weigh-in, baseline values were not fully restored, underscoring the fragile equilibrium between LEA, physiological recovery, and functional capacity in combat sports preparation.

The ecological validity of this study—rooted in real-world training, dietary practices, and performance testing closely replicating pre-competition conditions—adds practical relevance to the findings. These insights contribute to the ongoing discourse on safe weight management in athletes and reinforce the need for evidence-based, individualized guidelines to safeguard health while maintaining competitive performance. Further research should prioritize sex-specific physiological responses and long-term adaptations to LEA in combat sport contexts.

## Data Availability

The original contributions presented in the study are included in the article/Supplementary Material, further inquiries can be directed to the corresponding author.
